# Senescence-related genes analysis in breast cancer reveals the immune microenvironment and implications for immunotherapy

**DOI:** 10.18632/aging.205544

**Published:** 2024-02-14

**Authors:** Hua Zhong, Lijie Chang, Shengbin Pei, Yakun Kang, Lili Yang, Yifan Wu, Nuo Chen, Yicheng Luo, Yixiao Zhou, Jiaheng Xie, Yiqin Xia

**Affiliations:** 1 Department of Breast Surgery, The First Affiliated Hospital, Nanjing Medical University, Jiangsu Province Hospital, Nanjing, China; 2 Department of Neonatal Intensive Care Unit, The Second Affiliated Hospital, Anhui Medical University, Hefei, China; 3 Department of Burn and Plastic Surgery, The First Affiliated Hospital of Nanjing Medical University, Jiangsu Province Hospital, Nanjing, China

**Keywords:** senescence, immune microenvironment, immunotherapy, risk signature, breast cancer

## Abstract

Despite the advent of precision therapy for breast cancer (BRCA) treatment, some individuals are still unable to benefit from it and have poor survival prospects as a result of the disease’s high heterogeneity. Cell senescence plays a crucial role in the tumorigenesis, progression, and immune regulation of cancer and has a major impact on the tumor microenvironment. To find new treatment strategies, we aimed to investigate the potential significance of cell senescence in BRCA prognosis and immunotherapy. We created a 9-gene senescence-related signature. We evaluated the predictive power and the role of signatures in the immune microenvironment and infiltration. *In vitro* tests were used to validate the expression and function of the distinctive critical gene ACTC1. Our risk signature allows BRCA patients to receive a Predictive Risk Signature (PRS), which may be used to further categorize a patient's response to immunotherapy. Compared to conventional clinicopathological characteristics, PRS showed strong predictive efficacy and precise survival prediction. Moreover, PRS subgroups were examined for altered pathways, mutational patterns, and possibly useful medicines. Our research offers suggestions for incorporating senescence-based molecular classification into risk assessment and ICI therapy decision-making.

## INTRODUCTION

According to recent estimates, the prevalence of BRCA among women in all nations is extraordinarily high [1]. The typical radical mastectomy with radiotherapy and chemotherapy has been replaced in recent decades by a comprehensive multimodal approach that includes endocrine therapy and targeted therapy in addition to surgery [2]. However, these emerging therapies are still ineffective for some patients with BRCA because of the disease’s high degree of heterogeneity [3]. Recent developments in immune checkpoint antagonist therapy for gastric cancer and renal carcinoma have rekindled the passion for using these methods to treat and prevent BRCA [4]. However, the Food and Drug Administration (FDA) has only approved palivizumab and atezolizumab as immunotherapies for BRCA [5]. Therefore, better prognostic methods and biomarkers are urgently required to diagnose and treat BRCA accurately.

Cell senescence, a permanent state of cell cycle arrest and a significant feature of biological aging, was initially described by Hayflick in serially passaged human fibroblasts in culture [6, 7]. Senescent cells, although not proliferating, have metabolic and transcriptional activities, especially the release of inflammatory cytokines with autocrine, paracrine, and endocrine activities, and thus can influence their microenvironment [8]. A growing amount of research suggests that excessive and abnormal accumulation of senescent cells in tissues can negatively impact regenerative capacity and create a pro-inflammatory environment conducive to tumor initiation and progression [9]. Senescent fibroblasts have been shown to promote the growth of malignant cells in the breast, prostate, and skin tumors [10–12]. Senescent tumor cells also create a cytokine barrier that protects non-senescent tumor cells from immune attack by secreting factors that are part of the senescence-associated secretory phenotype (SASP) [13].

Great attention has been paid to the relationship between BRCA and cell senescence. According to recent research, DBC1-mediated cell senescence is present in BRCA [14]. A novel glycosylated indolocarbazole derivative, LCS1269, suppresses BRCA by inducing senescence [15]. The factors IL-6 and IL-8 secreted by senescent cells promote angiogenesis and tumor vascularization, promoting BRCA tumor cell invasion [16]. Lapatinib (L) and Fulvestrant (F), the classic drugs for targeted anti-BRCA therapy, were found to induce cell senescence in BRCA [17]. Senescence is characterized by heterogeneity, which is becoming a key factor in drug resistance in triple-negative breast cancer treatment [18]. Ionizing radiation (radiotherapy) can inhibit tumor growth by causing premature senescence of breast stromal cells [19]. However, no current studies or data exist to elucidate the relationship between cell senescence and BRCA outcomes. Therefore, accurate analysis of prognostic genes in BRCA is crucial, and exploration of gene profiles related to cell senescence is also vital for BRCA.

In this article, we tried to figure out whether cell senescence has a tight connection with BRCA prognosis based on current research and databases. In addition, we wish to create a signature associated with cell senescence to determine BRCA patients’ prognosis. Eventually, we sought to assess the link between cell senescence and immunotherapy and chemotherapy to discover more novel therapeutic strategies.

## RESULTS

### Differentially expressed genes associated with cell senescence

**Figure 1:**
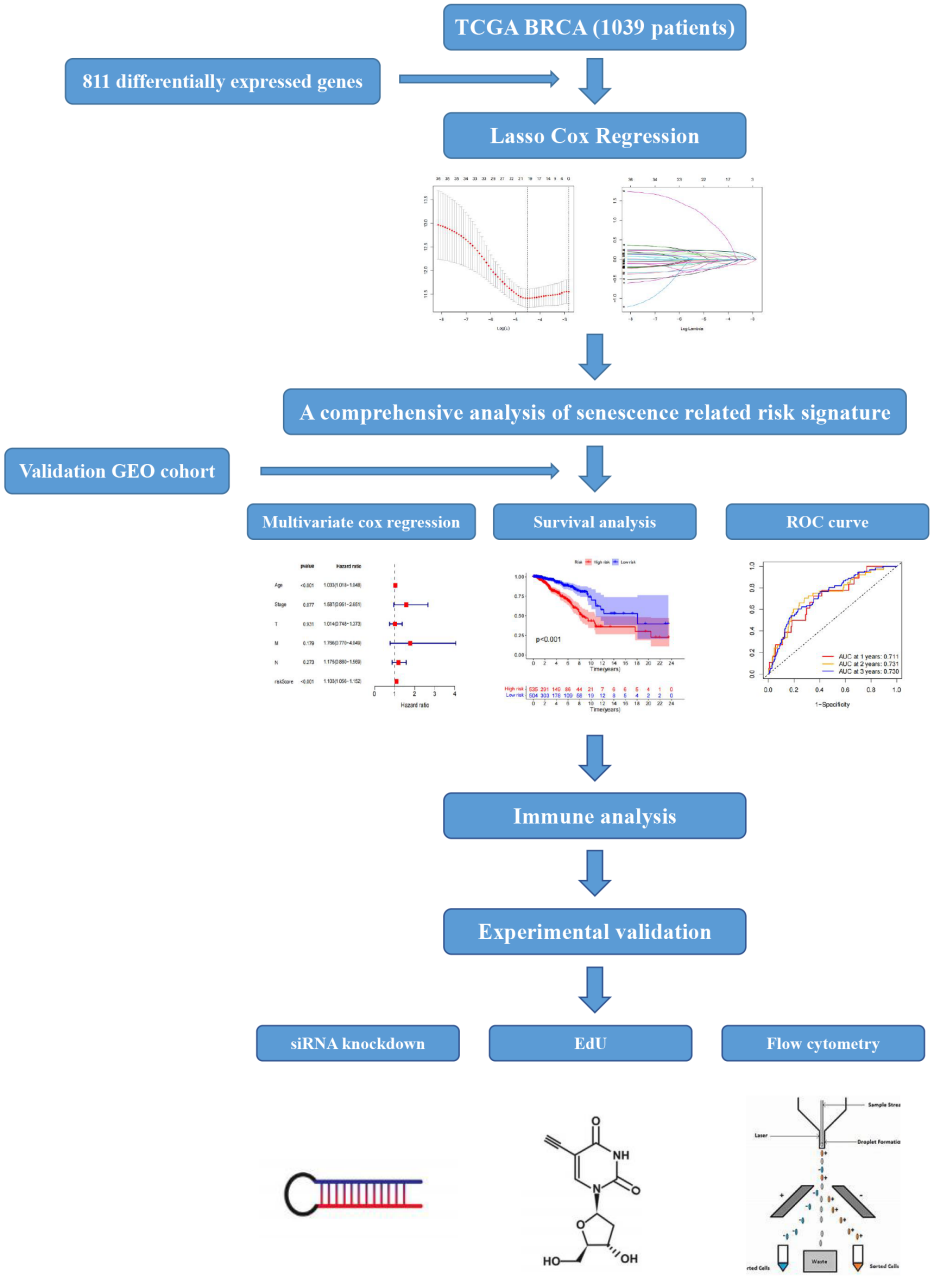
The flowchart of our study.

**Figure 2:**
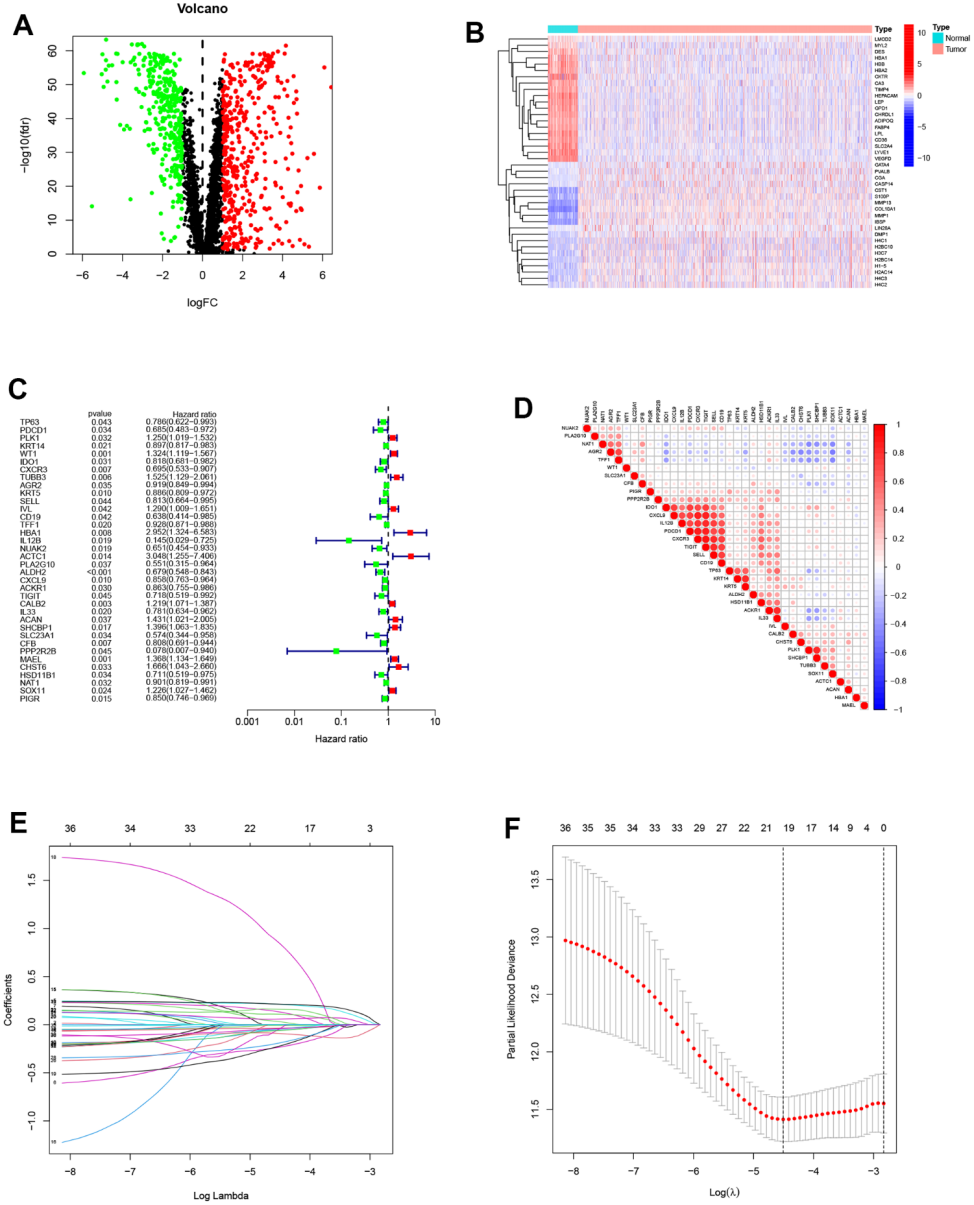
**Construction of the prognostic model.** (**A**) Differentially expressed genes between normal and tumor groups. (**B**) The top 20 differentially expressed genes were shown in the heatmap. (**C**) The forest plot of prognosis-related genes contained the Hazard Ratio (HR) and its 95% confidence interval. (**D**) Correlations between the prognosis-related genes. (**E**, **F**) The potential prognostic genes were subjected to LASSO-Cox regression in the training cohort to generate a prognostic risk signature.

Figure 1 displays the flow-process diagram of our study. Our inclusion criteria were mentioned above. Initially, we obtained the mRNA expression matrix from the whole expression matrix. 3604 genes in the TCGA database out of 4834 cell senescence-related genes obtained from the GENECARDS (https://www.genecards.org/) were extracted. Setting p-value < 0.05 and logFC >1 as the criteria, we got 811 differentially expressed genes shown in the volcano plot (Figure 2A). The heatmap shows the top 20 differentially expressed genes (Figure 2B).

### Establishment and verification of a 9-gene prognostic risk signature

Patients were randomly separated into the training cohort (520 samples) and the testing cohort (519 samples). Univariate Cox regression analysis was conducted on the diff-genes above, 36 genes potentially related to patients’ prognosis were screened out (Figure 2C). The associations of the 36 significant genes are shown in Figure 2D. Through Lasso analysis and Multivariate Cox regression analysis, we eventually established a 9-gene PRS (Figure 2E, 2F). The risk score can be calculated with the formula:


$$\begin{array}{l} \text{Riskscore}=0.299363285740934*\text{WT}1\text{-}\\ 0.409143279362624*\text{IDO}1\text{-}\\ 0.0828025876100934*\text{KRT}5+\\ 0.28610371540123*\text{IVL-}\\ 0.095153608202848*\text{TFF}1+\\ 1.22248770210134*\text{ACTC}1+\\ 0.319887428900399*\text{SHCBP}1\text{-}\\ 0.418057169642484*\text{SLC23A}1+\\ 0.240939666499152*\text{MAEL}. \end{array}$$

**Figure 3:**
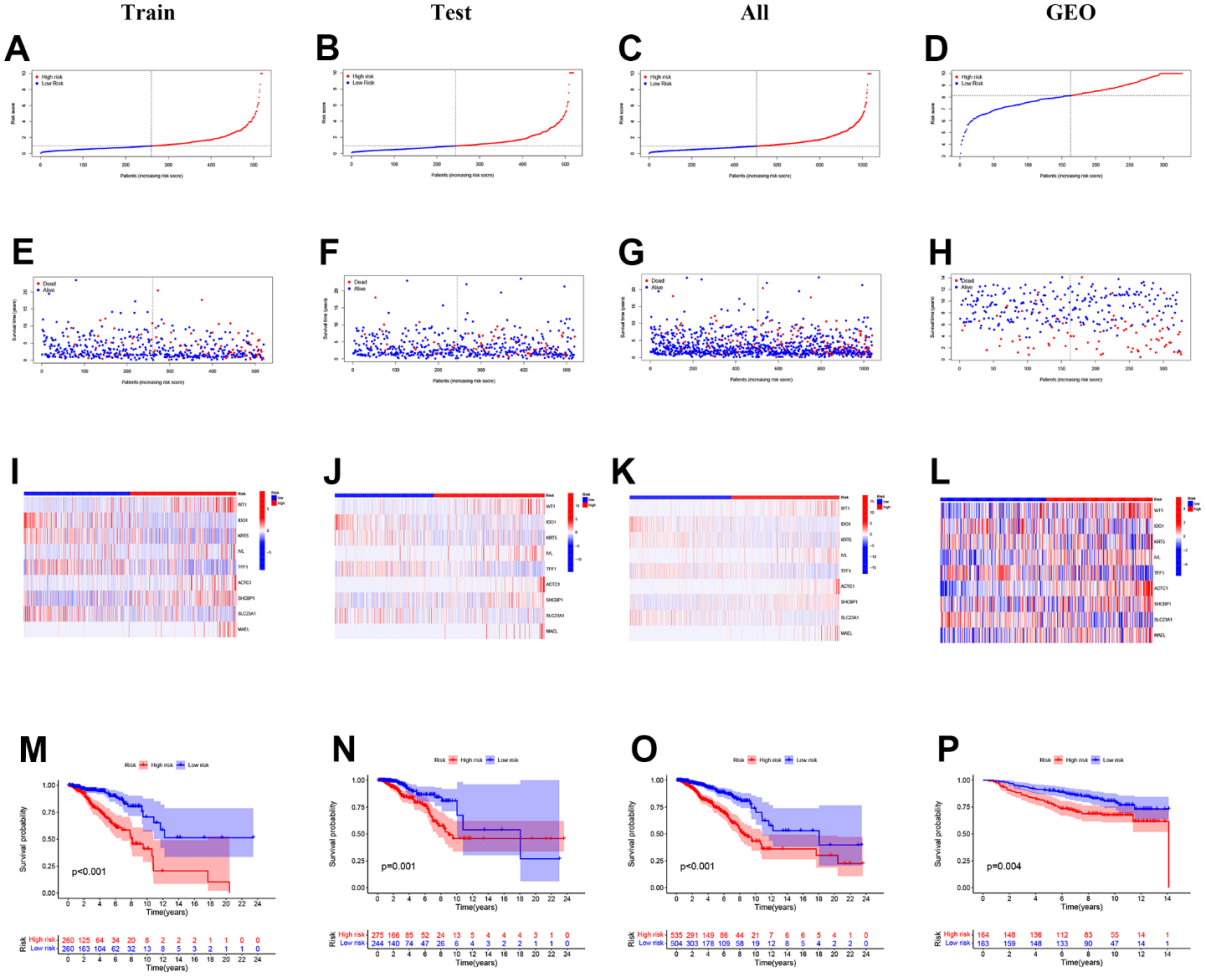
**The distribution of the risk scores, outcome status, and gene profiles of the gene signature in the training, testing, all, and GEO cohort were shown.** (**A**–**L**) The risk group successfully predicted the outcomes of the patients in both the training and validation cohorts, with significantly more events found in the high-risk group. (**E**–**H**) In the 9-gene risk signature, genes ACTC1, WT1, IVL, SHCBP1, and MAEL showed higher expression in the high-risk group. (**I**–**L**) The survival analysis is based on the prognostic model. (**M**–**P**) To further test the confidence of the risk model, a survival analysis was performed between the high-risk group and low-risk group among the training cohort, the testing cohort, all cohort, and the GEO cohort.

Figure 3A–3D shows the distribution of the calculated risk scores. In Figure 3E–3H, no matter which cohort, more death events can be discovered in the high-risk group, demonstrating that our PRS successfully predicted patients’ outcomes. Gene expression patterns were displayed in the heatmap (Figure 3I–3L). In the 9-gene PRS, genes ACTC1, WT1, IVL, SHCBP1, and MAEL showed higher expression in the high-risk group. To testify to the credibility of the 9-gene PRS, we conducted a survival analysis. The findings showed that the overall survival rate of patients in the training, testing, and entire cohort declined more sharply in the high-risk group than in the low-risk group (Figure 3M–3O). Similar trends were observed in the external validation GEO cohort (Figure 3P).

**Figure 4:**
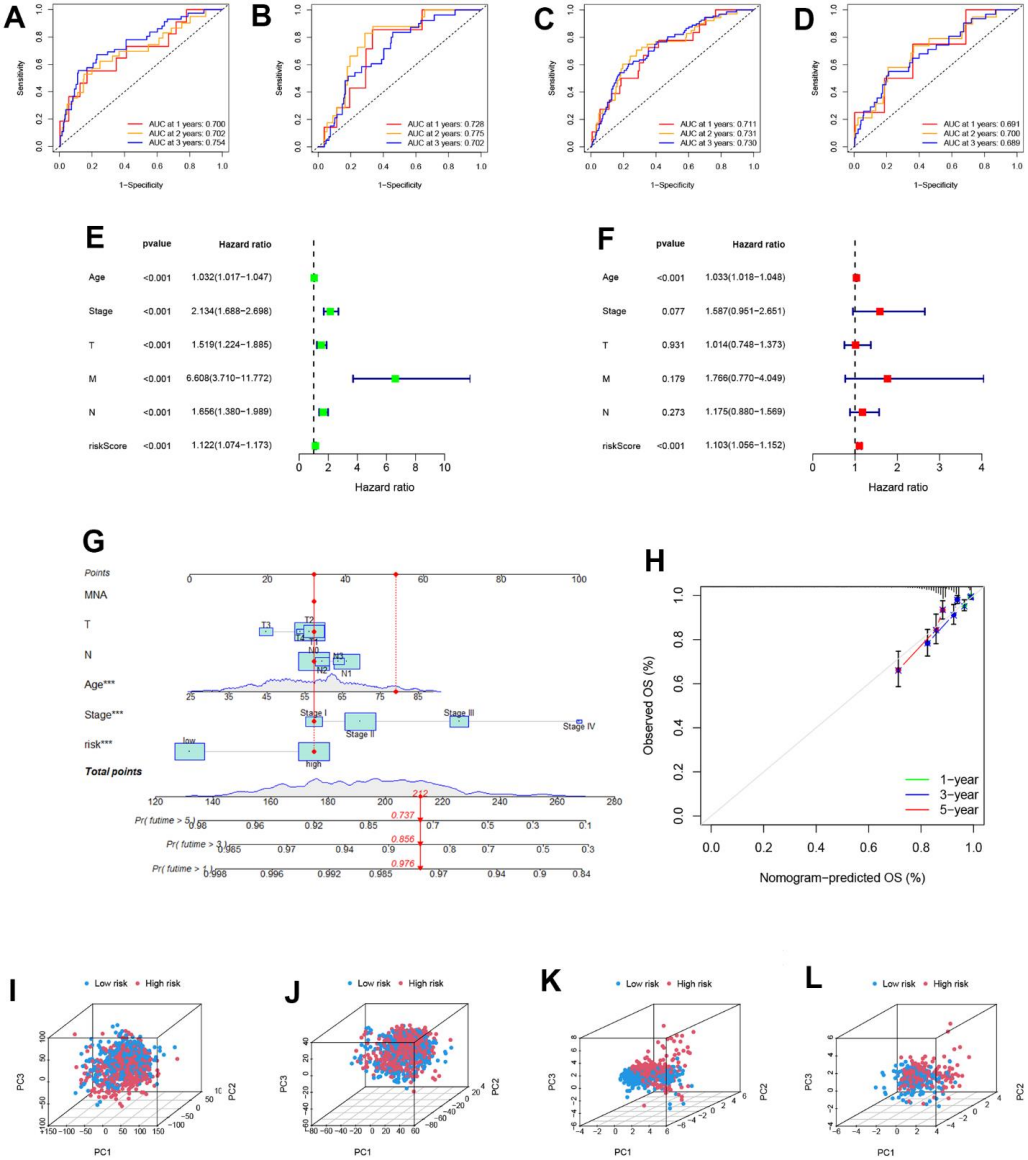
**Identification of the reliability of the model.** (**A**–**D**) The ROC curve of patients’ survival over different years in the training, testing, all, and GEO cohort showed that the model had a potent predicting ability. (**E**, **F**) Univariate and Multivariate Cox regression revealed that the risk score was an independent prognostic factor in BRCA patients. (**G**) Nomogram for predicting overall survival. (**H**) The calibration curves for 1-,3-, and 5-year OS. (**I**–**L**) The PCA 3D scatterplot of sample distribution is based on all genes, senescence-related genes, and model senescence-related genes in TCGA and GEO, respectively.

The areas under the 1, 2, and 3-year ROC curves (AUC) were: training cohort 0.700, 0.702, and 0.754, testing cohort 0.728, 0.775, 0.702, and all cohorts 0.711, 0.731, 0.730, respectively. All AUCs were over 0.7, demonstrating that our PRS effectively predicted BRCA Patients’ survival (Figure 4A–4C). The robustness and dependability of PRS are demonstrated by the AUC of the external validation GEO cohort, which was 0.691, 0.700, and 0.689 in 1, 2, and 3 years, respectively (Figure 4D). Clinical characteristics and risk score were analyzed using univariate and multivariate Cox regression to determine the independence of the risk score (Figure 4E, 4F). According to multi-Cox regression, only age and risk score can be seen as independent factors. The hazard ratios of risk scores were 1.122 and 1.103 in univariate and multivariate Cox regression, respectively (p<0.001). In light of the risk score, age, and other clinical characteristics, we drew a nomogram to predict the 1, 3, 5 years OS incidences, which shows 1, 3, 5 years OS rates of patient “TCGA-BH-A18K”. The calibration plot serves as evidence that the nomogram and the final result were in good agreement (Figure 4G, 4H). A three-dimension scatter plot of PCA analysis was drown to illustrate the PRS’s ability to classify BRCA samples. We found that the 9-gene PRS had superior discriminatory power and could better identify low-risk and high-risk populations (Figure 4I–4L).

### Biological function and pathway analyses

**Figure 5:**
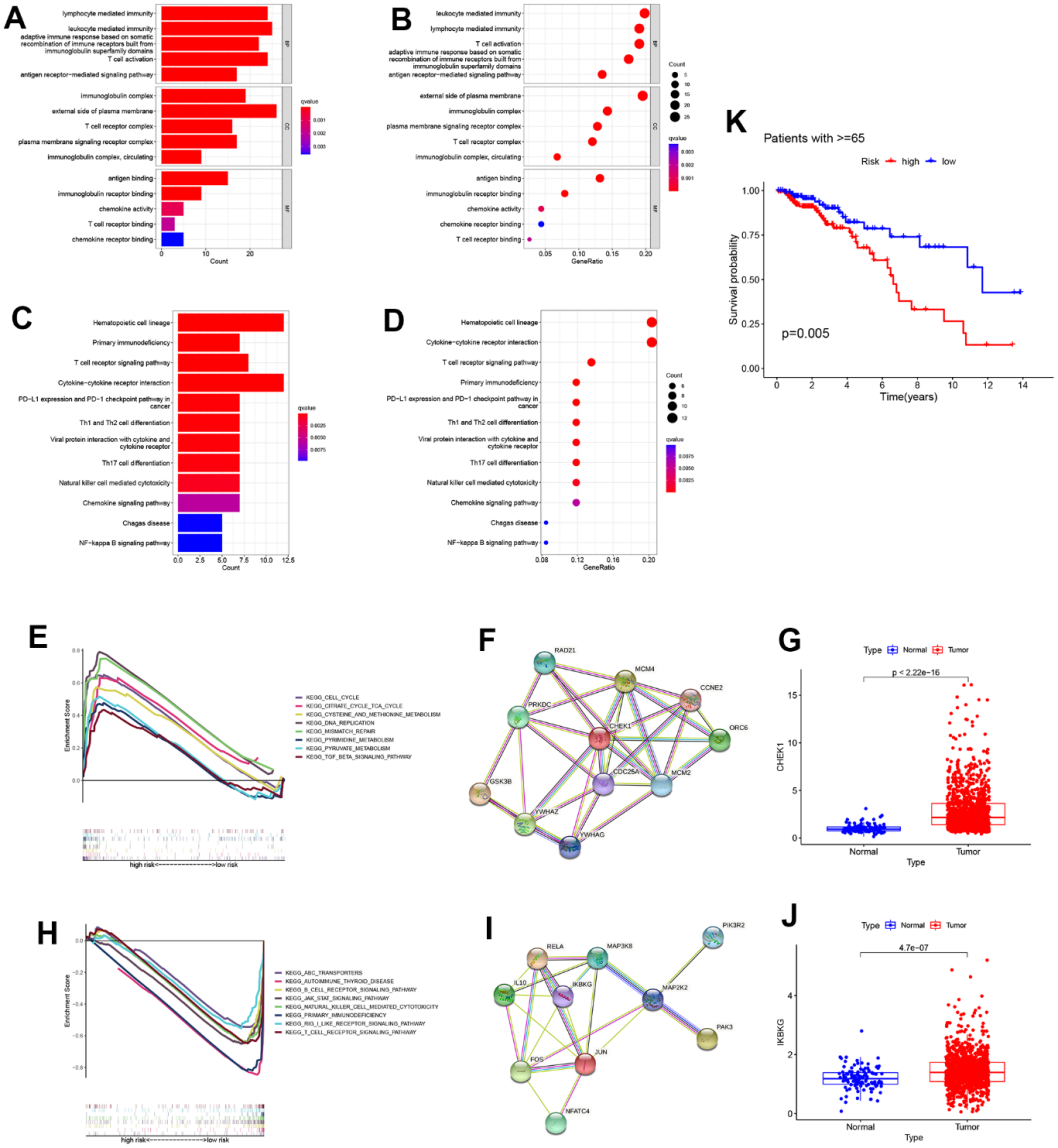
**Enrichment analysis in the TCGA all cohort.** (**A**, **B**) Bar plot and bubble plot of the Gene ontology (GO) enrichment. The results were divided into the biological process (BP), cell component (CC), and biological function (MF). The top five significant GO enrichment results are shown. (**C**, **D**) Bar plot and bubble plot of Kyoto Encyclopedia of Genes and Genomes enrichment. (KEGG) (**E**) Gene Set Enrichment Analysis (GSEA) revealed that the cell cycle was active in the high-risk group. (**F**) A protein-protein interaction network was generated to reveal interactions among the top ten genes involved in the “cell cycle”. (**G**) CHEK1 expression analysis between high and low-risk groups (*P*<0.05). (**H**) Gene Set Enrichment Analysis (GSEA) revealed that the “T cell receptor signaling pathway” was active in a low-risk group. (**I**) Representative genes involved in the “T cell receptor signaling pathway” were used to construct a protein-protein interaction network. (**J**) The expression profile of a critical checkpoint gene IKBKG was investigated in normal and tumor groups (*P*<0.05). (**K**) Survival analysis was performed on patients aged >=65.

We used bar charts and bubble charts to show the results of GO enrichment analysis, including: Molecular Function (MF), Cellular Component (CC), Biological Process (BP). The area of bubbles indicated the number of enriched genes. The color of the bubbles indicates enrichment significance. As is shown in the bar plot and bubble plot of GO enrichment analysis, the biological process includes lymphocyte-mediated immunity, T cell activation; molecular functions such as antigen binding, immunoglobulin receptor binding, T cell receptor binding; cell components like immunoglobulin complex and T cell receptor complex, indicating that enriched genes were strongly correlated with immune activities (Figure 5A, 5B). Similar conclusions can be drawn in the KEGG enrichment analysis. Immune pathways like the T cell receptor signaling pathway, PD−L1 expression, and PD−1 checkpoint pathway were concerned with the enriched genes (Figure 5C, 5D). To study the pathways regulated by cell senescence genes at a profound level, through GESA analysis between low and high-risk groups, the high-risk group associated with tumor (cell cycle, mismatch repair, and DNA replication). Here, we constructed a protein-protein interaction (PPI) network in the top ten related genes related to the ”cell cycle”. The key gene “CHEK1” expression was statistically different between tumor and normal groups (Figure 5E–5G). The low-risk group was correlated with immunity (B cell receptor signaling pathway, T cell receptor signaling pathway, and primary immunodeficiency). Similarly, a PPI network related to the “T cell receptor signaling pathway” was constructed, and we investigated the key gene “IKBKG” (Figure 5H–5J). Considering that senescence was closely related to aging, and the cell cycle pathway was more active in the high-risk group, a survival analysis was performed in patients age>=65. As is expected, high-risk patients have a worse prognosis (Figure 5K).

### Investigation of immune infiltration and microenvironment

**Figure 6:**
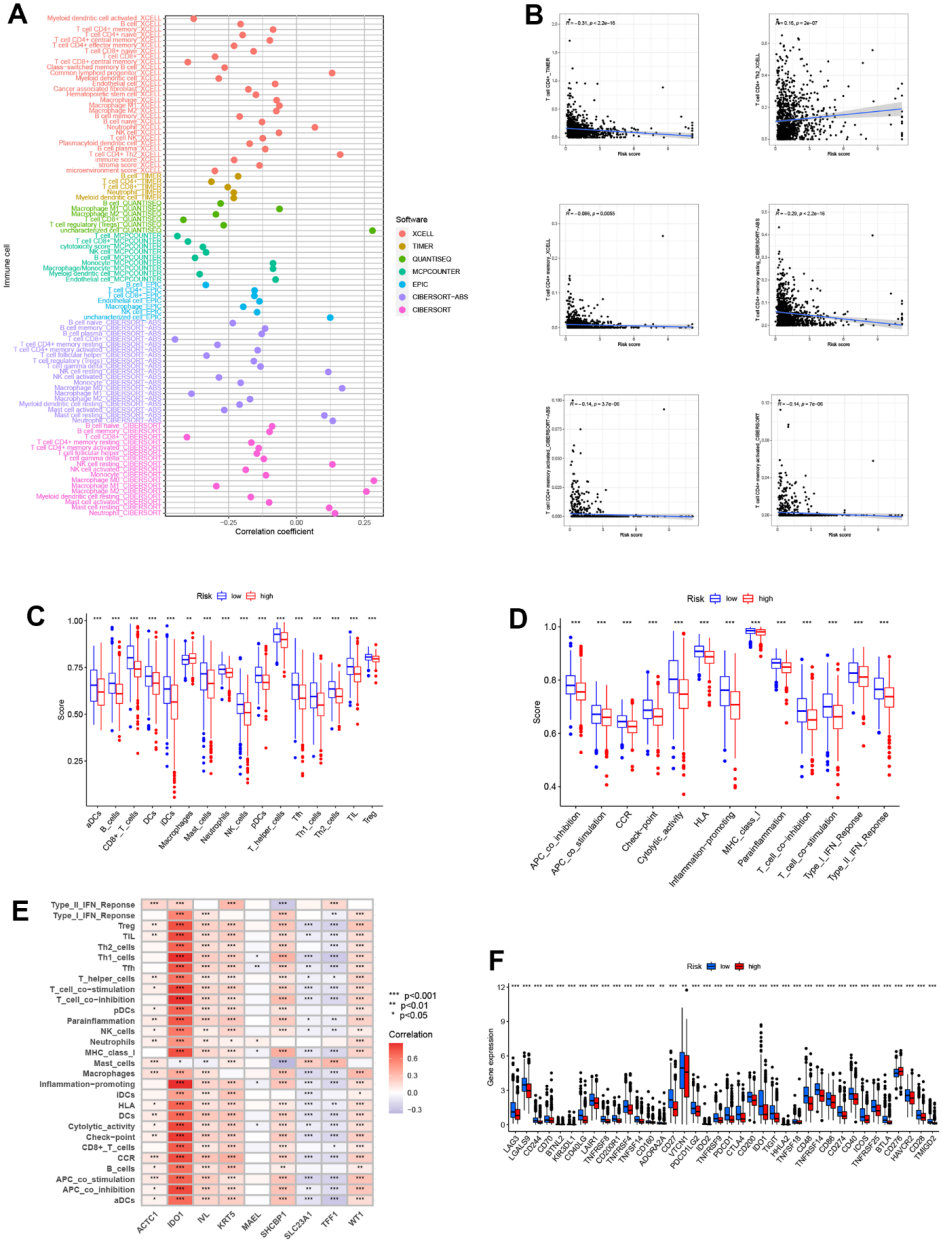
**Analysis related to immune between two groups.** (**A**) Immune cell bubble plot of risk groups. More immune cells were associated with the high-risk group. (**B**) Correlations between risk score and immune cell types. (**C**, **D**) ssGSEA scores of immune cells and immune functions between high and low-risk groups. ssGSEA scores were lower in the high-risk group. (**E**) Correlations between immune cells/functions and risk signature genes. (**F**) The immune checkpoint genes were expressed differently in the two groups, and the gene tended to be lower in the high-risk group (***P<0.001).

Based on the enrichment analysis above, we decided to perform immune-related analyses. Immune cells associated with risk groups on different platforms, such as TIMER, X-CELL, and CIBERSORT, were displayed. We could see that the vast majority of immune cells are inversely correlated with risk scores (Figure 6A). Then we selected some for detailed investigation (Figure 6B). Regarding total immune score, the score of patients in the high-risk group displayed a lower trend in immune cells and functions (Figure 6C, 6D). All these showed that lower levels of immune infiltration exist in the tumor microenvironment. In addition, we drew a heatmap of our model genes and immune cells or functions to demonstrate their relationships. ACTC1 was associated with nearly all immune factors (Figure 6E). Similarly, the immune checkpoint gene expressions showed a striking contrast in the two groups, and immune checkpoint gene expressions were commonly lower in the high-risk group (Figure 6F).

**Figure 7:**
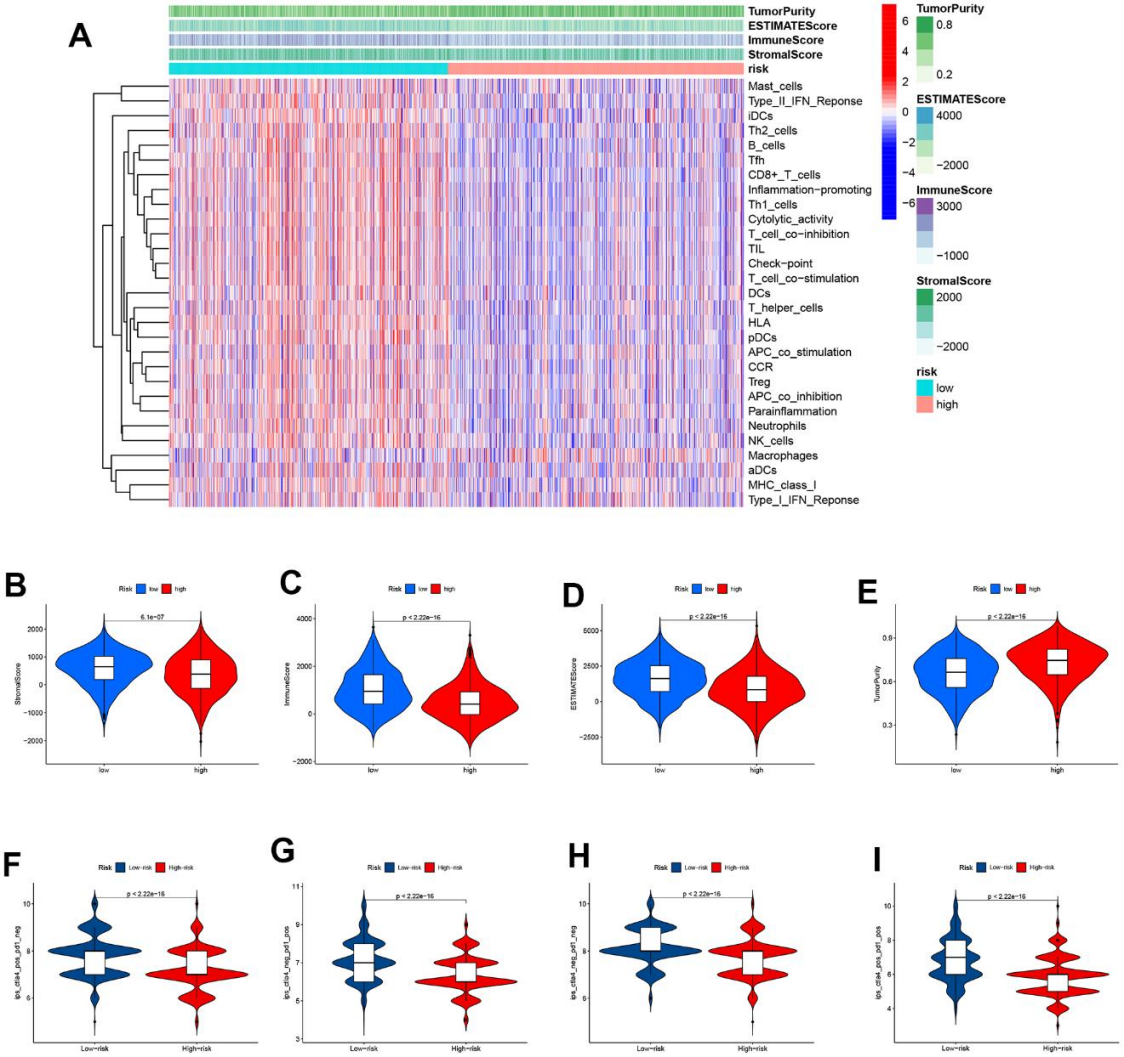
**Immunotherapy response analysis.** (**A**) A heatmap was drawn to illustrate the relationship between immune cell expression and stromal score, immune score, estimate score, and tumor purity. Immune cell expressions were lower in the high-risk group. (**B**) The stromal score was lower in the high-risk group than in the low-risk group (*P*<0.001). (**C**) The immune score was lower in the high-risk group than in the low-risk group (*P*<0.001). (**D**) The ESTIMATE score was lower in the high-risk group than in the low-risk group (*P*<0.001). (**E**) Patients in the high-risk group had higher tumor purity scores than those in the low-risk group (*P*<0.001). (**F**–**I**) The low-risk subtype has significantly greater IPS.

A heatmap was plotted to illustrate the correlation between immune cell/function and the scores above. The majority of immune cells are underexpressed in high-risk groups (Figure 7A). It is clear that the tumor purity score was higher in the high-risk group whereas the stromal score, immunological score, and estimation score were all lower in the high-risk group than they were in the low-risk group. The differences were statistically different (p<0.001). Similar to figure 7B–7E, the above scores all exhibited lower values in the high-risk group. This implies that in the high-risk group, there is not only lower expression of immune cells but also reduced immune infiltration, which is more likely to lead to tumor development. The use of Immunophenoscore (IPS) has the potential to aid in identifying patients who may be susceptible to immunotherapy. Our analysis of the Immunophenoscore (IPS) revealed that low-risk subtypes display higher IPS and blocker scores compared to high-risk subtypes. This study shows that low-risk individuals may respond to ICIs treatment more favorably and may gain more advantages (Figure 7F–7I).

### TMB and potential drug treatment

**Figure 8:**
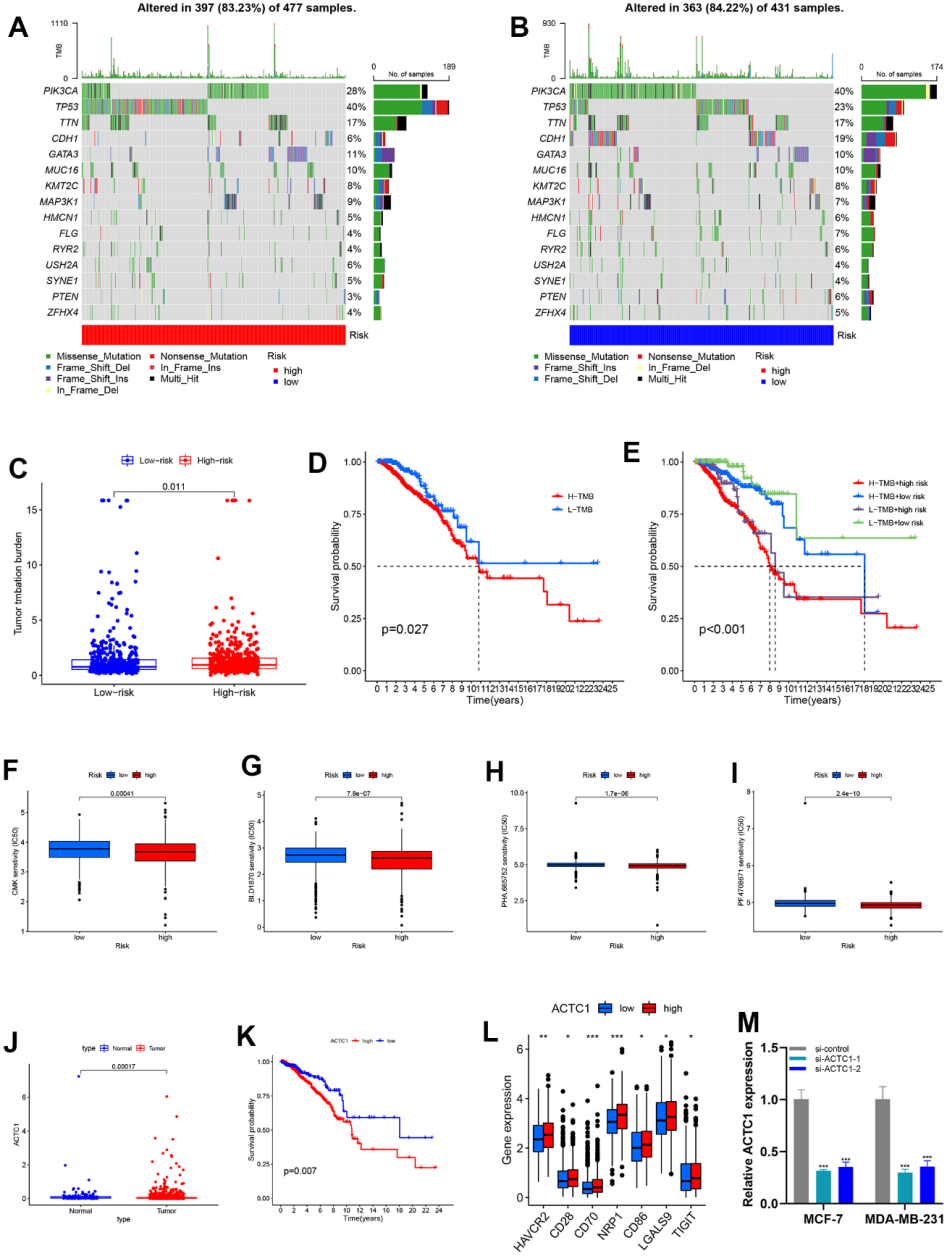
**Mutation analysis and sensitivity difference of antitumor drugs of BRCA.** (**A**, **B**) The landscape map of the top 15 genes with the highest mutation frequency, among which PIK3CA, TP53, and TTN genes are more prone to mutation. (**C**) Variance analysis revealed that high-risk groups have a higher tumor mutation burden. (**D**) K–M survival curves between the high-TMB and low-TMB sets. (**E**) K-M survival curves between H-H/H-L/L-H/L-L sets. (**F**–**I**) Boxplot showed the differential IC50 of our previously established high-risk and low-risk groups in the TCGA cohort. The high-risk group was more sensitive to antitumor drugs, including BI.D1870, CMK, PF.4708671, and PHA.665752. (**J**) Variance analysis of ACTC1 between tumor and normal groups. (**K**) Survival analysis of ACTC1 in high and low-risk groups. (**L**) Association of ACTC1 and immune checkpoints. (**M**) After ACTC1 siRNA transfection into MDA-MB-231 and MCF-7 cell lines, the ACTC1 expression level was significantly reduced.

Tumor mutation burden (TMB) data was acquired from TCGA. The top five mutated genes were PIK3CA, TP53, TTN, CDH1, and GATA3. TP53 mutation frequencies were significantly higher in the high-risk group than in the low-risk group (Figure 8A, 8B). Results showed that patients in the high-risk group had higher TMB (Figure 8C). As depicted in Figure 8D, a strong correlation exists between high TMB and poor survival results. Additionally, when patients were stratified into subsets, the H/H set exhibited a worse survival outcome (Figure 8E). Through drug sensitivity assessment, we found that BI.D1870, CMK, PF.4708671, and PHA.665752 showed a lower IC50 in the high-risk group, indicating that we may treat BRCA with these drugs at a lower dose (Figure 8F–8I).

### Analysis of ACTC1 in BRCA

Analysis of samples in TCGA demonstrated that ACTC1 expression was higher in tumor samples (Figure 8J), correlating with the result that patients with high ACTC1 expression had poor prognoses (Figure 8K). We could see that immune checkpoint expression was positively correlated with ACTC1 expression in BRCA (Figure 8L).

### The proliferation of BRCA cells went slow after ACTC1 knockdown

**Figure 9:**
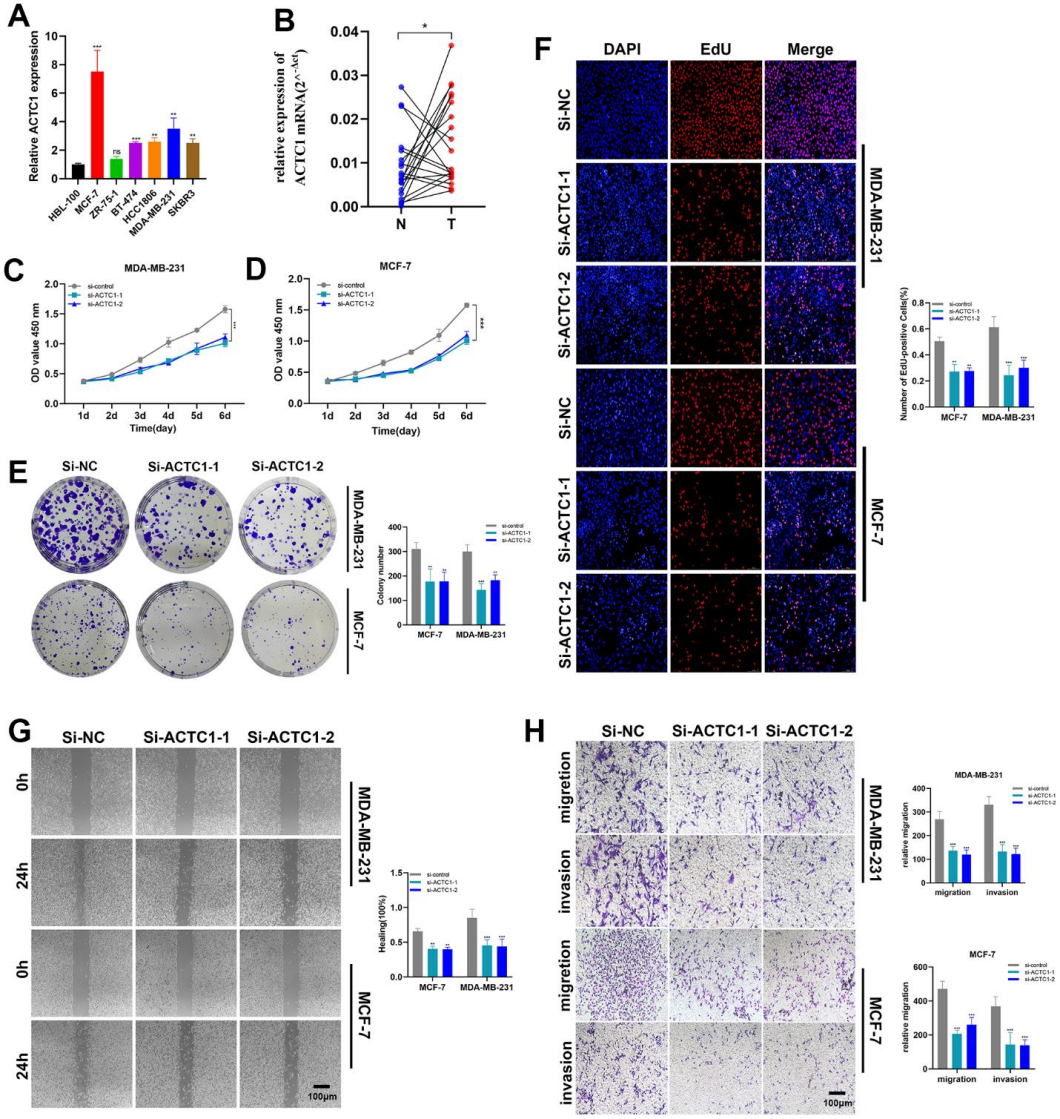
**ACTC1 knockdown *in vitro* experiment.** (**A**) RT-qPCR analysis was performed to verify the knockdown of ACTC1. (**B**) Expression analysis of ACTC1 in 20 pairs of BRCA tissue samples. (**C**, **D**) CCK-8 experiments. The activity of MDA-MB-231 and MCF-7 cell lines decreased significantly. (**E**) The cloning ability of MDA-MB-231 and MCF-7 cell lines decreased significantly. (**F**) EdU test. After ACTC1 knockdown, the proliferation ability of MDA-MB-231 and MCF-7 cell lines decreased significantly. (**G**) Healing test. After ACTC1 knockdown, the migration ability of MDA-MB-231 and MCF-7 cell lines decreased significantly. (**H**) Transwell assay. The migration and invasion abilities of MDA-MB-231 and MCF-7 cell lines were significantly decreased (*P<0.05, **P < 0.01, P***<0.001).

To further illustrate the function of ACTC1, *in vitro* experiments were conducted. Firstly, RT-qPCR analysis was performed to verify the knockdown of ACTC1. In all seven BRCA cell lines, ACTC1 expression was remarkably higher compared with normal controls. Among them, MDA-MB-231 and MCF-7 were the highest (Figure 9A). Figure 8M shows that after ACTC1 siRNA transfection into the two highest cell lines, the expression level of ACTC1 was significantly reduced. Upon subjecting twenty pairs of BRCA tissue samples from our hospital to the same validation procedure, we observed analogous expression patterns in clinical samples (Figure 9B). Notably, the ACTC1 gene exhibited high expression levels in 60% of the tissue pairs analysed. These results reinforce our initial hypotheses. In the CCK-8 assay, the proliferation ability of MDA-MB-231 and MCF-7 cell lines was substantially reduced after ACTC1 knockdown (Figure 9C, 9D). Figure 9E illustrates that the ability of MDA-MB-231 and MCF-7 cell lines to form colonies was markedly reduced upon ACTC1 knockdown. Moreover, the EdU assay demonstrated a notable decline in the proliferation rates of these cell lines following ACTC1 knockdown, indicating that ACTC1 may promote proliferation (Figure 9F).

### Knockdown of ACTC1 weakens the migration and invasion of BRCA cells

In comparison to the disordered siRNA control, the results of the scratch assay and transwell assay in Figure 9G, 9H showed that ACTC1 knockdown significantly decreased the ability of the MDA-MB-231 and MCF-7 cell lines to migrate and invade. Further, *in vivo* animal experiments also confirmed the results (Supplementary Figure 1). By extrapolating from the data, ACTC1 may exert a pivotal influence in fostering cellular migration and invasion, evinced by the statistically significant disparities with a p-value beneath 0.05.

### ACTC1 knockdown induced down-regulation of CD8+T cells

**Figure 10:**
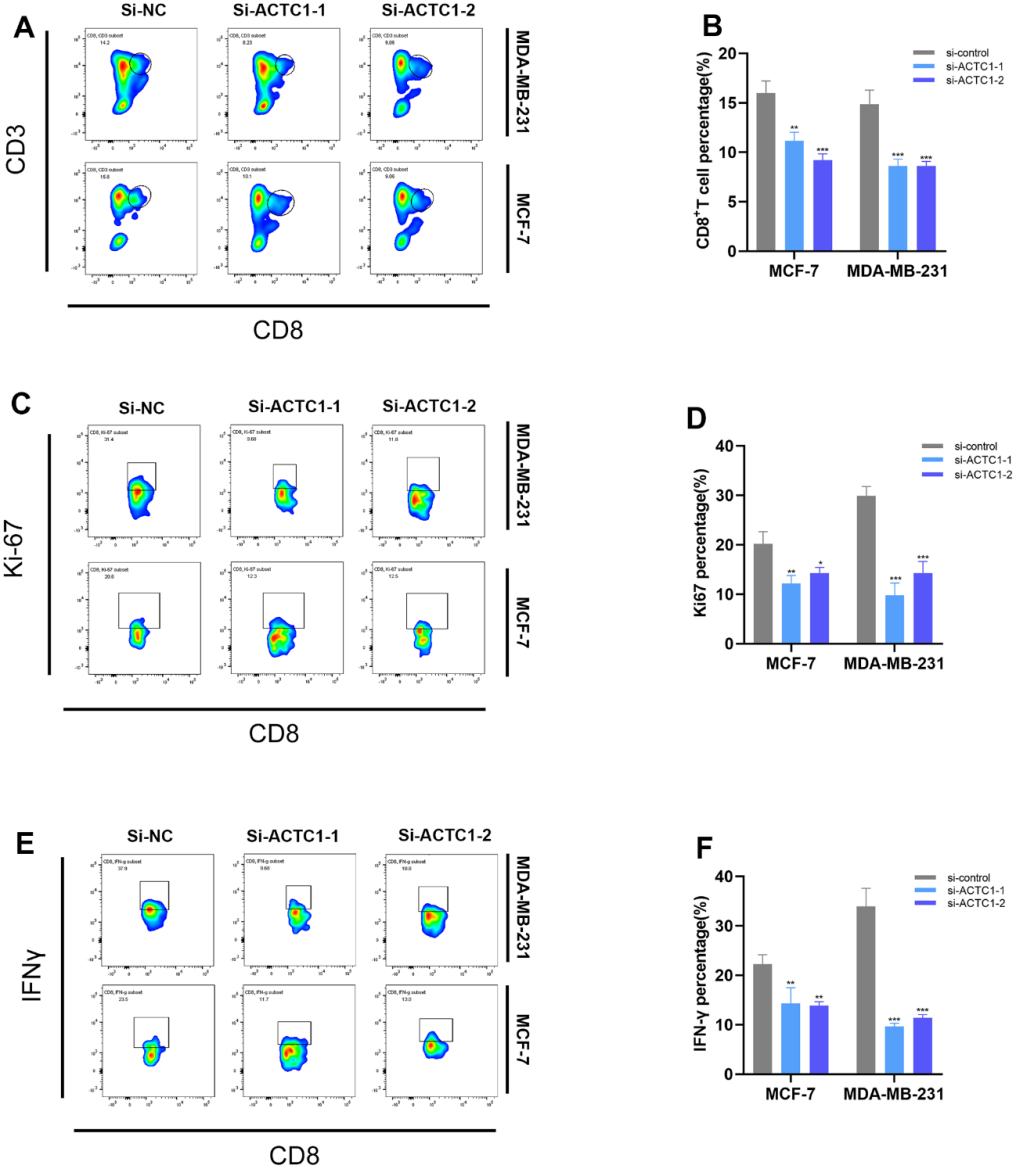
**Flow cytometry.** Representative plots (**A**) and a representative histogram (**B**) of the percentages of CD8+ T cells. Representative plots (**C**) and a representative histogram (**D**) of the percentages of Ki67. Representative plots (**E**) and a representative histogram (**F**) of the percentages of IFN-γ.

To investigate the effect of ACTC1 on immune cells, we co-cultured treated breast cancer tumor cells with PBMC and selected the corresponding antibodies to detect the percentage and activity of CD8+T cells. According to flow cytometry findings, the proportion of peripheral CD8+T cells (Figure 10A), proliferative BRCA cells (Ki67) (Figure 10C), and cytokine interferon-γ (IFN-γ) (Figure 10E) were significantly decreased when ACTC1 knockdown BRCA cells were co-cultured with PBMC, suggesting that ACTC1 knockdown may inhibit the activation of the immune system. The histogram illustrated that the differences were statistically significant. (Figure 10B refers to CD8+T cells, Figure 10D refers to Ki67, and Figure 10F refers to IFN-γ).

## DISCUSSION

BRCA is now cancer with the highest occurrence globally, and because of its heterogeneity, precision medicine is now used to diagnose and treat BRCA patients [1]. BRCA, previously deemed as possessing feeble immunogenicity, has now advanced into the realm of immunotherapy, owing to the efficaciousness of the treatment. In the age of individualized medicine, BRCA immunotherapy has proven difficult. The intricate and dynamic interplay between cancer cells and the immune system is in a state of perpetual evolution [20]. Unlike targeted therapy and endocrine therapy, there are no therapeutic indicators for immunotherapy. PD-L1 status, TMB, immunogenomic characteristics, and TILs have all been utilized as predictors of BRCA immunotherapy response in the past. However, none has enough data to be used as a stratification factor [21]. Consequently, additional research into the molecular mechanisms and prognostic biomarkers for BRCA may offer a chance to recognize BRCA subgroups and enhance precision-focused BRCA treatment. To bridge these gaps, we constructed and validated a nine-gene prognostic model by combining cellular senescence genes with the TCGA data cohort. Compared to several common clinicopathological features, our prognostic model shows strong and superior predictive power.

Cell senescence denotes a condition of irreversible cell cycle arrest, typified by anomalous cell structure and morphology and concomitant release of numerous pro-inflammatory factors that exert a substantial influence on the microenvironment [6, 8]. Senescent cells may support tumor suppression by recruiting cytotoxic T cells and NK cells [22]. However, how senescent cells interact with tumor immune infiltration and their value in assessing tumor immune infiltration and clinical outcome have not been reported in BRCA. Therefore, the study of cell senescence-related genes is of paramount significance for analyzing the tumor microenvironment of BRCA, and the prognosis and immunotherapy response of patients. Meanwhile, revealing how cell senescence affects TME could provide a window into how we can effectively improve the immunosuppressive environment through senescence therapies. In this study, we constructed an independent prognostic risk signature based on cell senescence-related DEG to comprehensively analyze the clinical significance and role of senescence-related genes in the immune microenvironment of BRCA, which may help clinicians to develop personalized immunotherapy strategies for patients.

To begin with, 4834 cell senescence-related genes acquired from the GENECARDS were extracted. Then, using nine genes related to senescence, we created a unique survival risk signature that worked well in validation cohorts from the TCGA and GEO. A nomogram that incorporates prognostic risk signature and clinicopathological factors was also developed. The PRS exhibited superior precision and discerning ability in prognosticating OS compared to conventional features like TNM. Our findings imply that low PRS patients benefit more from immunotherapy. Hence, there may be a very strong connection between immunotherapy and cell senescence. Studies have shown that almost all senescent cells release a wide variety of biologically active molecules, including cytokines, chemokines, growth factors, and proteases, called SASP [23]. SASP can directly affect the innate and adaptive immune systems, as it promotes the recruitment, differentiation, and/or activation of natural killer cells, macrophages, monocytes, neutrophils, and T lymphocytes [24]. Senescent cells can increase the activation of monocytes and their differentiation into macrophages by secreting granulocyte colony-stimulating factor (G-CSF) and macrophage colony-stimulating factor (M-CSF), respectively [25]. Senescent fibroblasts in aged skin secrete MCP-1, which attracts pro-inflammatory monocytes [26]. In addition, senescent cells can promote the proliferation of the CD38+ macrophage population, which leads to a decline in NAD+ in humans [27]. Senescent cells may also attenuate T-cell responses in older adults by promoting the secretion of T-cell-suppressing prostaglandin E2 (PGE 2), suggesting that cell senescence is also associated with decreased adaptive immunity [28]. Our findings also revealed that the immune infiltrating capacity differs among the 16 immune cells between the two groups of different PRS patients. As anticipated, patients with a low PRS manifested heightened vulnerability to tumor immune responses relative to their high PRS counterparts. Intriguingly, macrophages solely exhibited augmented scores in the high PRS group. Conversely, other immune cells, including CD8 T cells and others, exhibited diminished scores within the low PRS group. Correspondingly, the immune function and immune checkpoints in the high-PRS group demonstrated a decreasing inclination relative to their low-risk counterparts. This observation may suggest that it hardly activates important regulatory immunity, which could plausibly account for the dismal prognosis associated with the high-risk group. Our observations revealed that the low-risk group exhibited elevated IPS scores, correlated with better response to Immune Checkpoint Blockade (ICB) treatment. At the same time, our pathway enrichment analysis of the high- and low-risk groups showed that immune pathways, such as primary immune regulation, were enriched in the low-risk group. However, additional experiments are needed to demonstrate which cell senescence-related genes regulate the two different immunizations.

Immunotherapy research for BRCA mainly focuses on vaccines, chimeric antigen receptor T cell (CAR-T) therapy, and immune checkpoint inhibitors [29, 30]. ICIs have received the most attention, and many exploratory studies have been carried out clinically and fundamentally [31]. However, the effect of ICIs as a single treatment strategy in BRCA could be better, and its combination therapy is the main direction of current treatment [32]. We surmise that a novel and promising therapeutic approach could involve ICI-directed intervention in conjunction with cell senescence. Empirical evidence supports this conjecture and documents that cisplatin elicits pervasive cell senescence contingent on the cytosolic DNA sensor cyclic GMP-AMP synthase (CGAS) in high-grade serous ovarian cancer (HGSOC). This, in turn, triggers the secretion of CCL5, CXCL10, and IL6 by senescent HGSOC cells, thereby instigating the recruitment of immune effectors to the TME [33]. In conclusion, MPB tumors treated with cisplatin exhibited increased sensitivity to PD-1 by activating cell senescence. CDK4/6 inhibitors induce senescence and, when combined with immunotherapy, reduce melanoma expansion in a vivo mouse model [34]. Multiple findings suggest that chemotherapy or radiation therapy can stimulate the tumor environment, inducing cell senescence to support restored ICI sensitivity [35, 36]. Similarly, cell senescence alone may not eliminate tumor cells without additional measures. However, treatments that enhance immune activity may enhance the antitumor activity of recruited cells, highlighting the importance of combining senescence-inducing agents and ICIs in treating cold tumors.

Systemic chemotherapy continues to be the cornerstone of BRCA management. However, the chemotherapy regimen chosen varies. From our assessment of chemotherapeutic agents, we ascertained that four drugs exhibited greater sensitivity towards high PRS. These drugs are not first-line BRCA chemotherapy drugs; further research is required to investigate their feasibility for treating BRCA. CMK is a kinase inhibitor of RSK2, which has been identified to promote cell senescence in many tumors and is a promising therapeutic target for triple-negative breast cancer (TNBC) [37]. PF.4708671, an effective cell-permeable S6K1 inhibitor that enhances tamoxifen-induced MCF-7 cell death [38]. PHA-665752 is a selective ATP-competitive c-Met inhibitor that inhibits the metastasis of liver cancer and is also a potential target for the treatment of TNBC [39]. BI-D1870 is an ATP-competitive inhibitor of the S6 ribosome. P90 ribosomal S6 kinase (RSK) inhibitor BI-D1870 has been shown to promote cell senescence and counteract trastuzumab resistance in many tumors [40]. If more data support this finding, future use of these four drugs may support precision therapy. We hope our model will help predict new drugs and identify new therapeutic targets.

Among the 36 signature genes, Cardiac α-Actin (ACTC1) was the gene with the highest HR in our constructed signature and showed high expression in BRCA cells. ACTC1 is closely related to cellular aging, and it has been reported that aging changes the gene expression of growth and remodeling factors in human skeletal muscle, including ACTC1 [41]. Numerous antecedent investigations have demonstrated the implication of ACTC1 in oncogenesis [42, 43]. *In vitro*, the depletion of ACTC1 impedes the migratory capacity of glioblastoma cells [44]. ACTC1 in BRCA has not been identified yet, and our cell tests showed that knocking down ACTC1 expression significantly reduced the viability, invasion, and migration ability of BRCA cells. By co-culture with immune cells, we found that the knockdown of ACTC1 reduced CD8+T cell activity and function, which contributes to the substantiation that ACTC1 is implicated in BRCA. Our investigation unveiled that ACTC1 represents a potential target in BRCA. More vivo and *in vitro* mechanistic experiments of ACTC1 should be carried out to illustrate its role in BRCA progress and immunotherapy.

In this study, the TCGA dataset was systematically divided into a training set and a validation set to construct our prognosis model. In order to prevent improper modeling methods due to personal preferences, we used the GEO external data set to validate the constructed model and select the best model based on its accuracy. Nonetheless, like other similar studies, our study inevitably has limitations. First, although we collected multiple independent cohorts, further validation in prospective studies is warranted. Second, although multiple genes included in the constructed model appear in many prognostic features of cancer, this suggests that they have consistent prognostic value. Their role in BRCA remains to be elucidated and more functional experimental validation is needed in the future. Finally, further clinical trials are needed to confirm the efficacy of BI.D1870, CMK, PF.4708671, and PHA.665752 in high-risk BRCA patients.

## CONCLUSIONS

Our study pioneered the construction of a prognostic model for BRCA using senescence-related genes. The senescence-related genes signature can adequately classify patients for prognosis and immunological assessment of patients with breast cancer. To analyze the differential expression of immune microenvironment, immune infiltration, and immune checkpoint in two groups of breast cancer patients, and further explore sensitive drugs for patients with poor prognosis, to provide an effective basis for precise treatment of breast cancer. It engenders novel concepts for the diagnosis, treatment, and prognosis of BRCA. In addition, uncovering how cell senescence affects the TME could provide opportunities to exploit the cell senescence process to reshape the immunosuppressive milieu effectively. Future basic medical research is expected to explore the immune microenvironment of BRCA further.

## MATERIALS AND METHODS

### Gathering of original data

The RNA sequence dataset for BRCA was gathered from the TCGA database https://portal.gdc.cancer.gov/. Patients who met the following criteria were included in our study. 1) BRCA patients; 2) mRNA expression and clinical data were complete; 3) follow-up time for more than 30 days. In all, 1039 patients were included for further study. We also downloaded RNA sequence data from the GEO database (GSE20685).

### Collection of genes associated with cell senescence

On the GENECARDS website (https://www.genecards.org/), Setting “Cell Senescence” as a keyword, 4834 genes in all were acquired. The downloaded genes with a correlation score >1 were included. Eventually, 3604 genes in the TCGA database were filtered out for our subsequent analysis.

### Development and implementation of a prognostic-related model

Initially, we performed differential analysis to screen for differentially expressed genes between tumor and normal samples. A heatmap and a volcano plot were drawn. Patients with detailed clinical information were stochastically partitioned into training and testing cohorts in an equal 1:1 proportion. Afterward, we utilized the R package “survival” to conduct a univariate cox regression analysis to explore prognostic-related genes further. These genes were followingly chosen to establish a cell senescence-related prognostic model by Lasso and the Multivariate cox regression algorithm. We have categorized the groups into high and low-risk based on the risk score that falls in the middle of the range. Risk scores were derived through the utilization of the scoring formula:


$$\text{risk}\;\text{score}=\sum_{i=1}^{n}\beta i\times Ei$$

(The risk coefficient is denoted by β, while the expression of each gene in the model is represented by E.).

### Identification of the established 9-gene risk signature

To evaluate the OS difference between the two risk groups. The Kaplan-Meier method was utilized for survival analysis. Patients were then divided into subgroups by clinical features for survival analysis. The ROC curve verified the prediction efficiency on a 1, 2, and 3-year basis. To determine the precision of the PRS, we assessed it using the concordance index (C-index). We also exerted univariate and multivariate Cox regression analysis on both groups to identify that risk score and other clinicopathological features were independent prognostic indicators for BRCA patients. A nomogram and a calibration curve were also constructed based on age, stage, and TMN. The PCA analysis was used to classify BRCA samples through cell senescence gene expression patterns. A three-dimension scatterplot was drown to display the spatial distribution of different samples. The analyses were based on “forestplot”, “survival”, “pec”, “glmnet”, “rms”, “survminer”, “timeROC”, “scatterplot3D”, and “limma” R packages. Further verification was performed on the testing and all cohort.

### Analysis of enrichment functional pathways

To examine the relevant biological process and pathways implicated in the two risk groups, GO and KEGG enrichment analysis was conducted. In this study, Gene Set Enrichment Analysis (GSEA) analysis was performed to explore KEGG pathways differences in gene function. Key genes were further evaluated. The R packages “clusterProfiler”, “org.Hs.eg.db”, “enrichplot”, “dplyr”, “ggpubr” were utilized for the above analysis.

### Immune landscapes associated with prognostic model

According to the results above, immune landscape exploration was conducted. All TCGA tumors’ profile infiltration estimates were downloaded from TIMER2.0 (http://timer.cistrome.org/). In this way, the immune infiltration level of BRCA patients was calculated. A heatmap was plotted to illustrate the relationship between model genes and immune cells/functions. The r package “ggpubr”, “limma”, “scales”, “ggtext”, “tidyverse”, “GSVA”, and “GSEAbase” were utilized. Utilizing the technique of Single Sample Gene Set Enrichment Analysis (ssGSEA), we endeavored to unravel any variations in the functionality of diverse immune cells or systems. We made comparisons about tumor microenvironment (TME) scores, tumor purity by R package “estimate”, and immune checkpoint activation between the two groups. Eventually, the Immunophenoscore (IPS) of BC patients in TCGA was calculated through The Cancer Immunome Atlas (TCIA) (https://tcia.at).

### Variations of the somatic mutations

We acquired TMB data of BRCA patients from the TCGA database. Subsequently, we calculated the TMB value, and a maftool was plotted to visualize the TMB level difference. Based on TMB median score, patients were categorized into two sets: one with a higher TMB score and the other with a lower TMB score. We then performed a survival analysis to explore the variance in survival rates between the two sets. Subsequently, high and low TMB sets were separated into subsets (H-H/H-L L-H/L-L) for further analysis.

### Explorations of clinical treatment for BRCA patients

Using the R package “pRRophetic”, we selected potentially effective drugs for BRCA treatment based on half-maximal inhibitory concentration’s (IC50) statistical significance (*P*<0.05) in high-risk and low-risk groups.

### Analysis of ACTC1 in BRCA

We observed that ACTC1 has the highest hazard ratio, so we selected it for follow-up research. We evaluated its expression between tumor and normal samples. Besides, survival analysis and immune cell analysis associated with ACTC1 were also performed.

### Collecting of tissue samples and culture of cell lines

Twenty pairs of samples (tumor tissue (T) and precancerous tissue (N)) were obtained from BRCA patients undergoing tumor resection between February and March 2021. The Cell Resource Center of the Shanghai Life Sciences Institute provided human BRCA cell lines (MDA-MB-231, HCC1806, ZR-75-1, MCF-7, SKBR3, BT-474) and human normal breast epithelial cell line (HBL-100), which were grown in DMEM or RPMI-1640 (Gibco BRL, USA). Cells were cultured at 37° C with 10% fetal bovine serum (Gibco BRL, USA), 100U/mL penicillin, and 100g/mL streptomycin in an atmosphere with 95% humidity and 5% CO_2_.

### Cell transfection

Ribobio created and manufactured an anti-ACTC1 small interfering RNA (siRNA) probe (Guangzhou, China). Lipofectamine 3000 was used for all transfections (Invitrogen, USA). In Supplementary Table 1, the siRNA sequences for ACTC1 are shown.

### RNA extraction and real-time polymerase chain reaction (RT-PCR)

TRIzol was used to extract total RNA from tissues or cell lines in accordance with the manufacturer’s instructions (15596018, Thermo). After that, the PrimeScriptTMRT kit was used to produce cDNA (R232-01, Vazyme). RT-PCR was performed using the SYBR Green Master Mix (Q111-02, Vazyme), and the expression levels were calculated using the 2^-ΔtΔt^ method. The level of mRNA GAPDH expression served to standardize each mRNA’s expression level. Detailed primer sequences may be found in Supplementary Table 1; Tsingke Biotech contributed all primers (Beijing, China).

### Cell counting kit-8 experiment (CCK-8)

At first, 5×10^3^ cells per well were seeded in the cell suspension in 96-well plates. All these cells were precultured. Following that, the plate was incubated with 10 mL of CCK-8 labeling solution (A311-01, Vazyme) in each well for 2 hours at 37° C in a dark environment. The enzyme-labeled meter (A33978, Thermo) was used to measure the cells’ absorbance at 450 nm in order to determine their health. Six measurements were made every 24 hours.

### Colony formation

We transfected 2×10^3^ cells per well in a 6-well dish. Before colonies became apparent, all cells were kept alive for a period of two weeks. The cells were fixed in 4% paraformaldehyde for 15 minutes after being rinsed twice with PBS, and then stained with Crystal Violet (Solarbio, China). The colonies in each well were then enumerated.

### EdU

Following the manufacturer’s instructions, 5-Ethynyl-2’-deoxyuridine (EdU) assay was conducted (Ribobio, China). We gave the cells two hours in a cell incubator before rinsing them with PBS and putting them in 4% paraformaldehyde for ten minutes with 0.5% Triton-X-100. The staining agent utilized was Apollo® fluorescent dye. The quantification of the cells undergoing proliferation was then conducted.

### Wound-healing assay

Transfected cells were seeded into 6-well plates and grown in a cell incubator until they were 95% confluent. Unattached cells and debris were carefully washed away after serum deprivation using PBS twice using a sterile 20μL plastic pipette tip. After capturing images of the scratch wounds at once and after two days, we utilized the ImageJ software to measure the width of the scratches.

### Transwell

Transwell assays were employed to investigate cell migration and invasion. Specifically, 2×10^4^ cells per well were incubated in a medium devoid of serum in the upper chamber. The 600μL full medium is kept in the lowest chamber. To test the capabilities of cell invasion and migration, the upper portion of the plate was either pre-coated with Matrigel solution (BD Biosciences, USA) or left untreated. Under a light microscope, cells were counted after being fixed with 4% PFA, dyed with 0.1% crystal violet (Solarbio, China), and fixed again.

### Statistical analysis and data handling

R (v4.1.3) and Strawberry Perl (v5.30.0) processed all our data and figures. Experimental data were processed by software Image J (v1.8.0) and Graphpad (v9.4.0).

### Flow cytometry

Transfected tumor cells and Peripheral Blood Mononuclear cells (PBMC) were co-cultured for two days and then stimulated by PMA. Then we added antibodies and incubated cells at room temperature for 30 minutes. Antibodies were as follows: CD3 (BD Biosciences), CD4 (BD Biosciences), CD8 (BD Biosciences), CD45 (BD Biosciences), IFN-γ (BD Biosciences) and Ki-67 (BD Biosciences). A BD FACSAria II cytometer was used for the analysis using flow cytometry (BD Biosciences). We subsequently analyzed the resulting data with the FlowJo software.

### Data availability statement

The original contributions presented in the study are included in the article Supplementary Material. Further inquiries can be directed to the corresponding authors. Publicly available datasets were analyzed in this study. This data can be found here: https://portal.gdc.cancer.gov/.

## Supplementary Material

Supplementary Figure 1

Supplementary Table 1
