# Supplementary material for: Senescence-related genes analysis in breast cancer reveals the immune microenvironment and implications for immunotherapy
Source: Aging (Albany NY). 2024 Feb 14;16(4):3531–53. doi: 10.18632/aging.205544 (PMC10929821; doi:10.18632/aging.205544)
Supplement: Supplementary Figure 1 [file aging-16-205544-s001.pdf]

SUPPLEMENTARY FIGURE

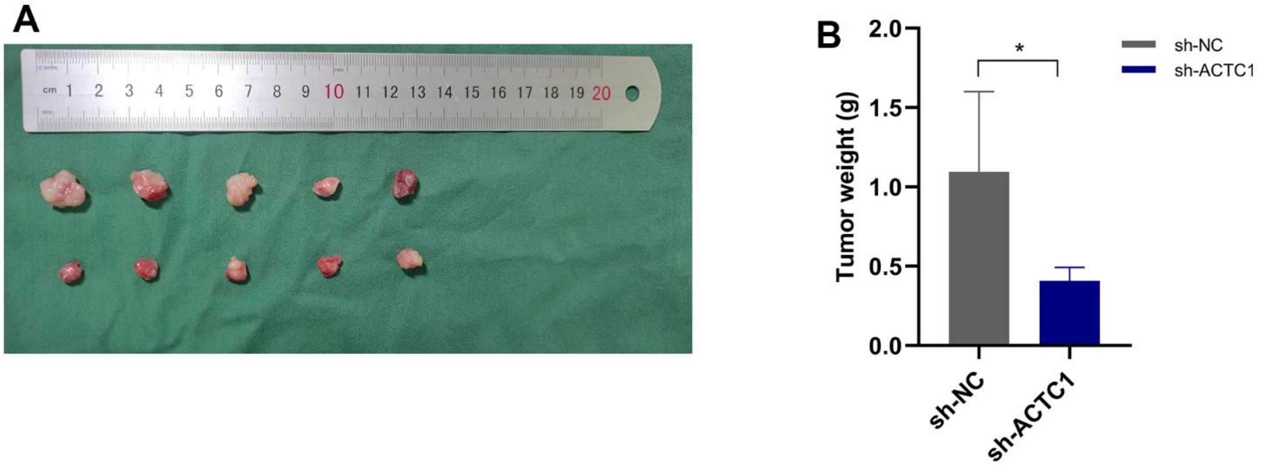

Supplementary Figure 1. ACTC1 function was verified *in vivo*. (**A**) Photographs of tumors obtained from the different groups of nude mice transfected with sh-NC and sh-ACTC1. (**B**) Knockdown of ACTC1 expression significantly inhibited breast cancer cell growth in nude mice and tumor weight was significantly reduced in the sh-ACTC1 group compared to that in the NC group.
