# Supplementary material for: Senescence-related genes analysis in breast cancer reveals the immune microenvironment and implications for immunotherapy
Source: Aging (Albany NY). 2024 Feb 14;16(4):3531–53. doi: 10.18632/aging.205544 (PMC10929821; doi:10.18632/aging.205544)
Supplement: Supplementary Table 1 [file aging-16-205544-s002.pdf]

SUPPLEMENTARY TABLE

Supplementary Table 1. Oligonucleotides used in research.

Oligonucleotides	Nucleotide sequence (5'-3')
**siRNA**	
Scramble control	GCUUCGCGCCGUAGUCUUA
Si-ACTC1-1	GGAUUAGCAAGCAAGAGUATTUACUCUUGCUUGCUAAUCCTT
SiACTC1-2	CUAGCACCAUGAAGAUUAATTUUAAUCUUCAUGGUGCUAGTT
**Primer**	
GAPDH	GGCCTCCAAGGAGTAAGACC (forward)<br>AGGGGAGATTCAGTGTGGTG (reverse)
ACTC1	GTACCCTGGTATTGCTGATCG (forward)<br>CCTCATCGTACTCTTGCTTGCT (reverse)
